# The Relationships between Body Composition and the Systemic Inflammatory Response in Patients with Primary Operable Colorectal Cancer

**DOI:** 10.1371/journal.pone.0041883

**Published:** 2012-08-03

**Authors:** Colin H. Richards, Campbell S. D. Roxburgh, Mark T. MacMillan, Sanad Isswiasi, Ewen G. Robertson, Graeme K. Guthrie, Paul G. Horgan, Donald C. McMillan

**Affiliations:** 1 University Department of Surgery, Glasgow Royal Infirmary, Glasgow, United Kingdom; 2 University Department of Radiology, Glasgow Royal Infirmary, Glasgow, United Kingdom; University of Colorado, United States of America

## Abstract

**Background:**

Weight loss is recognised as a marker of poor prognosis in patients with cancer but the aetiology of cancer cachexia remains unclear. The aim of the present study was to examine the relationships between CT measured parameters of body composition and the systemic inflammatory response in patients with primary operable colorectal cancer.

**Patient and Methods:**

174 patients with primary operable colorectal cancer who underwent resection with curative intent (2003–2010). Image analysis of CT scans was used to measure total fat index (cm^2^/m^2^), subcutaneous fat index (cm^2^/m^2^), visceral fat index (cm^2^/m^2^) and skeletal muscle index (cm^2^/m^2^). Systemic inflammatory response was measured by serum white cell count (WCC), neutrophil:lymphocyte ratio (NLR) and the Glasgow Prognostic Score (mGPS).

**Results:**

There were no relationships between any parameter of body composition and serum WCC or NLR. There was a significant relationship between low skeletal muscle index and an elevated systemic inflammatory response, as measured by the mGPS (p = 0.001). This was confirmed by linear relationships between skeletal muscle index and both C-reactive protein (r = −0.21, p = 0.005) and albumin (r = 0.31, p<0.001). There was no association between skeletal muscle index and tumour stage.

**Conclusions:**

The present study highlights a direct relationship between low levels of skeletal muscle and the presence of a systemic inflammatory response in patients with primary operable colorectal cancer.

## Introduction

Approximately 1 in 3 people in the United Kingdom will develop cancer during their lifetime [1]. Of these, almost half will experience a progressive involuntary weight loss with their disease, termed cancer cachexia. The degree of weight loss varies by tumour type but gastrointestinal tumours have a particularly high prevalence [2]. Indeed, it is estimated that up to half of patients with colorectal cancer have experienced weight loss by the time of presentation [3].

Cachexia has long been recognised as a marker of poor prognosis in cancer patients; associated with an increased risk of surgical complications [4], resistance to chemotherapy [5], [6], reduced quality of life [2] and decreased survival [7]–[9]. The clear link between weight loss, reduced performance status, impaired response to treatment and poor prognosis in such patients may be due to the preferential loss of skeletal muscle. It has been suggested that, although the loss of adipose tissue accounts for the majority of the weight loss, it is the loss of muscle which impacts upon morbidity and mortality [10]–[12]. This has led some to describe the phenomenon of cancer-related weight loss as ‘sarcopenia’; a term originally employed to describe the gradual loss of skeletal muscle seen with ageing. The aetiological factors responsible for these changes in body composition are unclear but previous observations indicate there may be an association with inflammation. Indeed, there is now evidence that the systemic inflammatory response, already recognized as a marker of poor prognosis in patients with gastrointestinal cancer [13], is associated with the cardinal features of cachexia [14], [15]. Previous work has demonstrated an association between systemic inflammation and a loss of lean tissue as measured using a total body potassium scanner [16] although such equipment is not routinely available, is unlikely to be useful in clinical practice and has been superseded by the advent of cross-sectional imaging.

The aim of the present study, therefore, was to examine the relationships between CT measured parameters of body composition and the systemic inflammatory response in patients with primary operable colorectal cancer.

## Methods

Patients with colorectal cancer who, on the basis of laparotomy findings and preoperative staging CT scan, were considered to have undergone potentially curative resection for colorectal cancer (Stage I – III) between January 1^st^ 2003 and December 31^st^ 2010 at Glasgow Royal Infirmary were identified from a prospectively maintained database. Of these, only patients with recorded height data and CT images taken preoperatively for diagnostic or staging purposes and stored in an electronic format suitable for image analysis were included in the study.

Patient height and weight was recorded from preoperative assessment health records and included only if documented within 30 days of CT scan. Patients were classified by body mass index (BMI) as underweight (BMI<18.5), normal weight (BMI 18.5–24.9), overweight (BMI 25.0–29.9) or obese (BMI>30) according to World Health Organisation (WHO) criteria. The tumours were staged according to the 5^th^ edition of the Tumour, Node and Metastases (TNM) classification [17]. Additional pathological data were taken from reports issued at the time of resection.

The systemic inflammatory response, as defined by a number of inflammation-based prognostic scores, has proven an important indicator of outcome in cancer patients [18]. In particular, the modified Glasgow Prognostic Score (mGPS) has been shown to reflect clinically relevant alterations in acute phase protein production (CRP and albumin) and is an established prognostic marker in colorectal cancer [19]. Preoperative systemic inflammatory response in the present study was thus assessed using three different measures (Table 1); (1) serum white cell count (WCC) [20], (2) neutrophil to lymphocyte ratio (NLR) [21] and (3) the modified Glasgow Prognostic Score (mGPS) [22].

**Table 1:** Systemic inflammation-based prognostic scores.

Prognostic score	Score
White cell count (WCC)	
WCC <8.5 (10^9^/l)	0
WCC 8.5–11.0 (10^9^/l)	1
WCC >11 (10^9^/l)	2
Neutrophil:Lymphocyte Ratio (NLR)	
NLR<5∶1	0
NLR≥5∶1	1
The modified Glasgow Prognostic Score (mGPS)	
C-reactive protein ≤10 mg/l and albumin ≥35 g/l	0
C-reactive protein ≤10 mg/l and albumin <35 g/l	0
C-reactive protein >10 mg/l	1
C-reactive protein >10 mg/l and albumin <35 g/l	2

**Figure 1:**
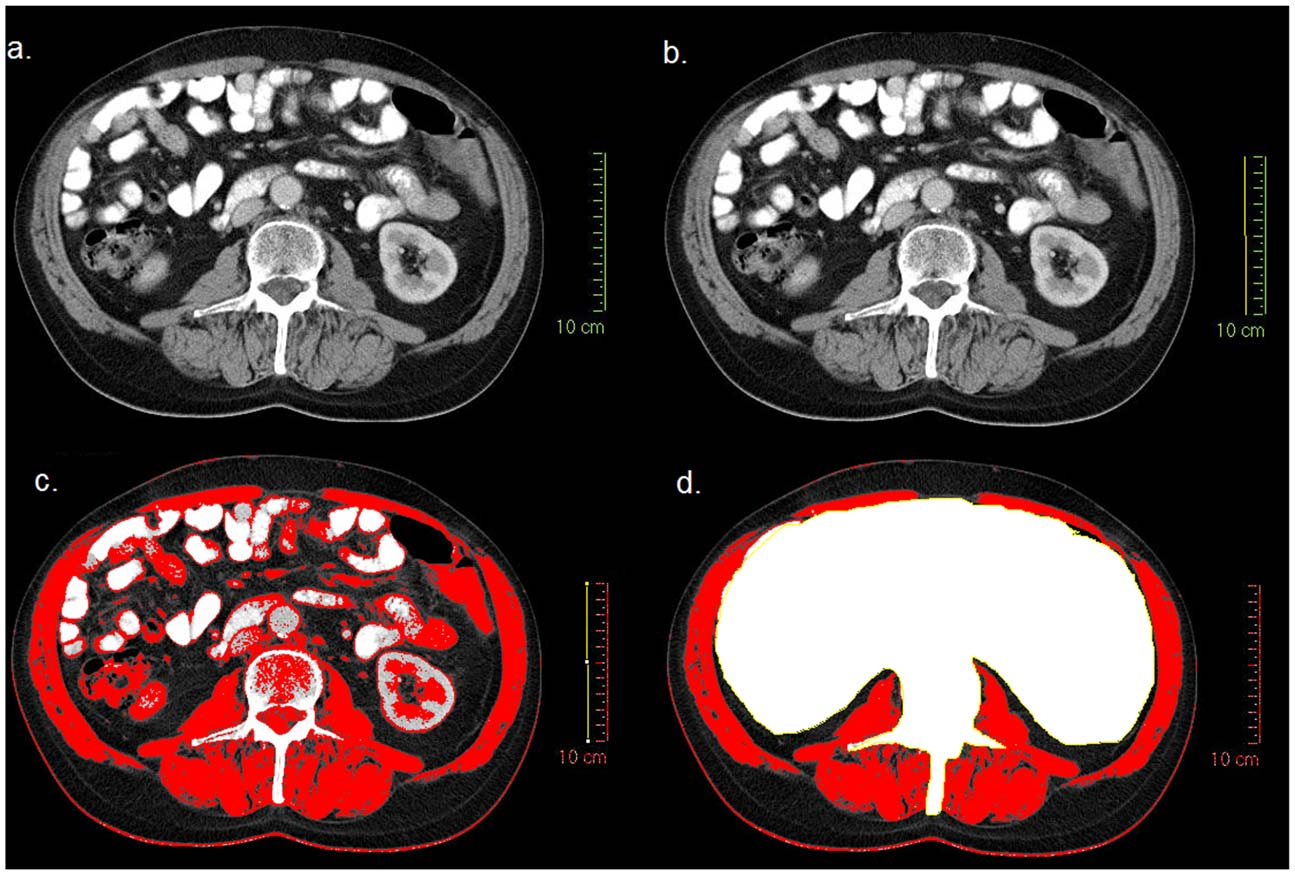
An example of CT image analysis using NIH ImageJ software. (a) the original CT image in JPEG format, (b) the scale is set using a known distance (10 cm) from the original CT image, (c) skeletal muscle thresholds (−29 to+150 HU) are applied, (d) the abdominal contents and L3 vertebrae are cropped and the skeletal muscle cross sectional area calculated in cm^2^.

The image analysis of CT scans was undertaken using medical imaging software. To test the reliability of different software packages, one commercially available program (Slice-O-Matic, version 4.3, Tomovision) and one governmental free-ware program (NIH ImageJ, version 1.44, http://rsbweb.nih.gov.ij/), were compared. Two trained investigators (CSDR and MTM) analysed a random sample of 50 cases using each of the software packages with the following results. (1) CSDR versus MTM using Slice-O-Matic software, mean difference of 4.51 cm^2^, limits of agreement −1.67 cm^2^ to 10.69 cm^2^, interclass correlation coefficient (ICC) = 0.977, (2) CSDR versus MTM using ImageJ software, mean difference of 1.52 cm^2^, limits of agreement −8.81 cm^2^ to 11.85 cm^2^, ICC = 0.987, (3) Slice-O-Matic versus ImageJ software, mean difference of 7.50 cm^2^, limits of agreement −13.63 cm^2^ to 28.64 cm^2^ ICC = 0.953. After establishing that both software packages provided reliable measurements, ImageJ was used for the entire cohort. Figure 1 provides an example of CT image analysis using NIH ImageJ software (Figure 1).

Total fat, subcutaneous fat, visceral fat and skeletal muscle cross-sectional areas (cm^2^) were measured at the level of L3 using standard Hounsfield unit ranges (adipose tissue: −190 to −30; skeletal muscle: −29 to +150) [23]. Each parameter was then normalized for patient stature, as is conventional for BMI, and designated as total fat index (cm^2^/m^2^), subcutaneous fat index (cm^2^/m^2^), visceral fat index (cm^2^/m^2^), skeletal muscle index (cm^2^/m^2^). To further test inter-observer agreement, each parameter was again measured independently by two investigators in a random sample of 50 cases (total fat index, ICC = 0.982; subcutaneous fat index, ICC = 0.992; visceral fat index, ICC = 0.955; skeletal muscle index, ICC = 0.987).

The authors confirm that this study was approved by the West of Scotland Research Ethics Committee, Glasgow with written informed consent obtained from all participants.

Body composition parameters are presented as mean values with standard deviation (SD) and are categorised into sex-specific tertiles (low/medium/high). Grouping of other variables was carried out using standard or previously published thresholds. Relationships between continuous and categorical variables were examined using *X* ^2^ linear-by-linear analysis, non-parametric tests and Pearson correlation coefficients (r) as appropriate. *P* values of less than 0.05 were considered statistically significant. Statistical analysis was performed using SPSS software (Version 18.0. SPSS Inc., Chicago, IL, USA).

## Results

**Figure 2:**
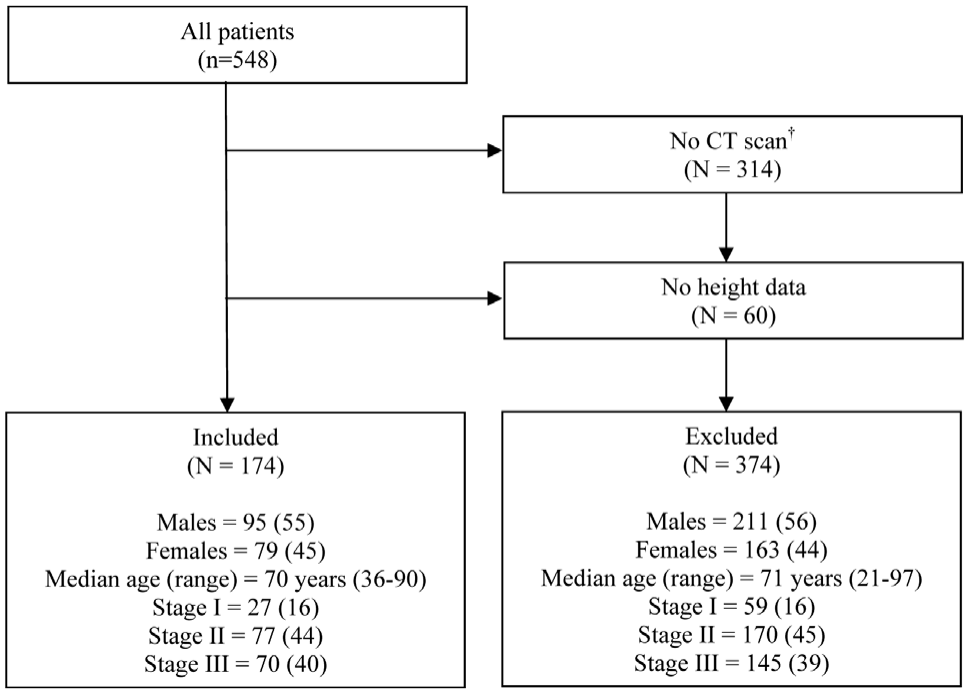
Flow chart representing the study selection process. ^*^ All patients undergoing potentially curative resection of colorectal cancer Janueary 1^st^ 2003 and December 31^st^ 2010. ^†^ No CT scan stored in an electronic format suitable for image analysis.

A total of 548 patients underwent potentially curative resection of colorectal cancer during the study period. Of these, 374 patients were excluded (314 patients did not have an electronic version of their CT scans available for image analysis and 60 patients did not have any height data recorded) and 174 patients were included. Figure 2 summarises the study selection process. Baseline clinico-pathological characteristics of the included cohort are shown in Table 2. Approximately one third of patients were 75 years or older with a similar number of males and females. The majority of patients had no evidence of a systemic inflammatory response prior to surgery. According to WHO BMI classification, 3% of patients were underweight, 36% normal weight, 33% overweight and 28% obese. The operations were carried out for colon cancer in 66% of cases and rectal cancer in 34%. Pathology reports classified 16% of the tumours as stage I, 44% as stage II and 40% as stage III (Table 2).

**Table 2:** Clinicopathological characteristics of patients with primary operable colorectal cancer.

Variable		N = 174 (%)
**Clinical variables**		
Age	≤64	51 (29)
	65–74	63 (36)
	≥75	60 (35)
Sex	Female	79 (45)
	Male	95 (55)
ASA grade*	1/2	77 (44)
	3/4	68 (39)
Presentation	Elective	165 (95)
	Emergency	9 (5)
Anaemia*	None	93 (53)
	Mild	50 (29)
	Severe	30 (17)
Smoking status*	Never	74 (43)
	Ex	64 (37)
	Current	33 (19)
**Inflammatory variables**		
White cell count (x10^9^/L)*	<8.5	112 (64)
	8.5–11	34 (20)
	>11	15 (9)
Neutrophil:lymphocyte ratio*	<5	118 (68)
	>5	34 (20)
mGPS	0	123 (71)
	1	20 (12)
	2	31 (18)
**Pathological variables**		
Tumour site	Colon	115 (66)
	Rectum	59 (34)
T stage	T 1/2	33 (19)
	T 3	94 (54)
	T 4	47 (27)
N Stage	N 0	105 (60)
	N 1	48 (28)
	N 2	21 (12)
TNM stage	Stage I	27 (16)
	Stage II	77 (44)
	Stage III	70 (40)
Venous invasion	Absent	77 (44)
	Present	97 (56)
Differentiation	Well/mod	163 (94)
	Poor	11 (6)
Lymph nodes retrieved	>12	130 (75)
	<12	44 (25)

* Missing values: ASA (n = 29), anaemia (n = 1), smoking (n = 3), white cell count (n = 13), neutrophil:lymphocyte ratio (n = 22).

The body composition parameters of the patients are shown in Table 3. There were no sex differences in BMI. Females had significantly more total fat (150.3 cm^2^/m^2^ versus 124.1 cm^2^/m^2^, p<0.001) and subcutaneous fat (104.4 cm^2^/m^2^ versus 73.7 cm^2^/m^2^, p<0.001) while males had significantly more skeletal muscle (46.2 cm^2^/m^2^ versus 36.9 cm^2^/m^2^, p<0.001). These differences justified the use sex-specific tertiles in the study i.e. data relating to body composition is thus corrected for sex (Table 3).

**Table 3:** Body composition parameters of patients with primary operable colorectal cancer.

Parameter	Male		Female		*p* *
	value	N (%)	value	N (%)	
Body mass index (kg/m^2^)					
Mean (SD)	27.7 (6.8)		26.9 (6.2)		0.59
Range	18.5–64.5		14.5–47.6		
Underweight	<18.5	1 (1)	<18.5	5 (6)	
Normal weight	18.5–24.9	33 (35)	18.5–24.9	30 (38)	
Overweight	25.0–29.9	37 (39)	25.0–29.9	20 (25)	
Obese	>30	24 (25)	>30	24 (30)	
Total fat index (cm^2^/m^2^)					
Mean (SD)	124.1 (52.2)		150.3 (58.6)		<0.001
Range	38.1–309.7		29.5–318.2		
Sex-specific tertile “Low”	38.0–101.0	32 (34)	29.5–130.5	27 (34)	
Sex-specific tertile“Medium”	101.0–134.5	32 (34)	130.5–177.5	27 (34)	
Sex-specific tertile “High”	134.5–310.0	31 (32)	177.5–318.5	25 (32)	
Subcutaneous fat index (cm^2^/m^2^)					
Mean (SD)	73.7 (37.5)		104.4 (44.6)		<0.001
Range	24.4–231.4		14.9–207.9		
Sex-specific tertile “Low”	24.0–58.5	32 (34)	14.5–85.5	27 (34)	
Sex-specific tertile“Medium”	58.5–73.5	32 (34)	85.5–129.5	27 (34)	
Sex-specific tertile “High”	73.5–231.5	31 (32)	129.5–208.0	25 (32)	
Visceral fat index (cm^2^/m^2^)					
Mean (SD)	50.4 (21.8)		45.9 (22.9)		0.13
Range	10.8–134.9		5.9–114.4		
Sex-specific tertile “Low”	10.5–40.5	32 (34)	5.5–37.5	27 (34)	
Sex-specific tertile“Medium”	40.5–55.5	32 (34)	37.5–50.5	27 (34)	
Sex-specific tertile “High”	55.5–135.0	31 (32)	50.5–114.5	25 (32)	
Skeletal muscle index (cm^2^/m^2^)					
Mean (SD)	46.2 (8.6)		36.9 (7.8)		<0.001
Range	26.9–68.8		24.8–72.2		
Sex-specific tertile “Low”	26.5–42.0	32 (34)	24.5–32.5	27 (34)	
Sex-specific tertile“Medium”	42.0–49.5	32 (34)	32.5–39.0	27 (34)	
Sex-specific tertile “High”	49.5–69.0	31 (33)	39.0–72.5	25 (32)	

* Mann-Whitney U test.

The relationships between parameters of body composition and measures of the systemic inflammatory response in patients with primary operable cancer are shown in Table 4. There were no relationships between any parameter of body composition and serum WCC or NLR. However, there was a significant relationship between an elevated mGPS and a low skeletal muscle index (p = 0.001) (Table 4).

**Table 4:** The relationships between parameters of body composition and measures of the systemic inflammatory response in patients with primary operable colorectal cancer.

Inflammatory response	Body mass index		Total fat index		Subcutaneous fat index				Visceral fat index		Skeletal muscle index	
	(kg/m^2^)		(cm^2^/m^2^)		(cm^2^/m^2^)			(cm^2^/m^2^)			(cm^2^/m^2^)	
	under/norm/over/obese	*p* *	low/med/high	*p* *	low/med/high	*p* *		low/med/high		*p* *	low/med/high	*p* *
WCC												
<8.5	4/43/39/26		39/39/34		41/35/36			38/44/30			39/37/36	
8.5–11	0/10/7/17		5/14/15		7/11/16			9/8/17			8/14/12	
>11	0/5/6/4	0.08	6/4/5	0.34	3/8/4	0.18		4/6/5		0.15	7/6/2	0.51
NLR												
<5	3/38/42/35		34/44/40		36/41/41		38/38/42				40/38/40	
>5	0/15/7/12	0.94	14/10/10	0.28	13/11/10	0.41	11/15/8			0.44	9/17/8	0.85
mGPS												
0	3/41/46/33		41/46/36		39/45/39		39/47/37				35/41/47	
1	1/5/5/9		4/4/12		6/4/10		5/5/10				7/7/6	
2	2/17/6/6	0.09	14/9/8	0.76	14/9/8	0.40	15/7/9			0.50	17/11/3	0.001

* *X* ^2^ linear-by-linear analysis.

WCC = white cell count.

NLR =  neutrophil;lymphocyte ratio.

mGPS =  modified Glasgow Prognostic Score.

**Figure 3:**
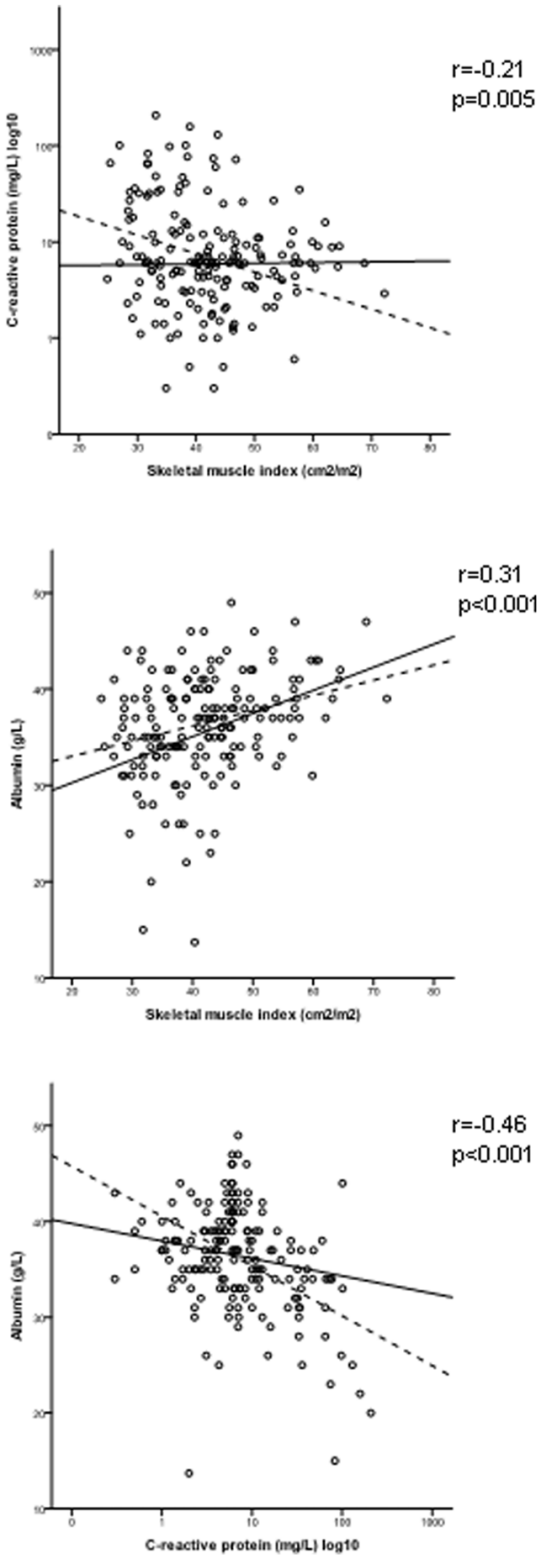
Scatterplots of the associations between C-reactive protein, albumin and skeletal muscle index. Fit lines are shown for male (**^____^**) and female (––-) patients. r = Pearsons correlation coefficient for all patients.

To further examine this relationship, absolute values of C-reactive protein and albumin were correlated with each parameter of body composition. With regard to C-reactive protein, there were no relationships with total fat index, subcutaneous fat index or visceral fat index but there was a significant negative correlation with skeletal muscle index (r = −0.21, p = 0.005). With regard to albumin, there were no relationships with total fat index or subcutaneous fat index but there were significant positive correlations with visceral fat index (r = 0.18, p = 0.02) and skeletal muscle index (r = 0.31, p<0.001). Scatterplots demonstrating these correlations are shown in Figure 3.

The relationships between skeletal muscle index and clinicopathological characteristics of the patients are shown in Table 5. There were significant associations between a low skeletal muscle index and increasing age (p<0.001) and presence of anaemia (p = 0.029). There were no associations between skeletal muscle index and any of the tumour-related variables (Table 5).

**Table 5:** The relationship between skeletal muscle index and clinico-pathological characteristics in patients with primary operable colorectal cancer.

Variable		Skeletal muscle index (cm^2^/m^2^)			*p* *
		Low	Medium	High	
		(n = 59)	(n = 59)	(n = 56)	
Age	≤64	8 (16)	14 (27)	29 (57)	
	65–74	22 (35)	24 (38)	17 (27)	
	≥75	29 (48)	21 (35)	10 (17)	<0.001
ASA grade	1/2	26 (34)	26 (34)	25 (32)	
	3/4	22 (32)	23 (34)	23 (34)	0.84
Presentation	Elective	53 (32)	58 (35)	54 (33)	
	Emergency	6 (67)	1 (11)	2 (22)	0.11
Anaemia	None	25 (27)	30 (32)	38 (41)	
	Mild	22 (44)	17 (34)	11 (22)	
	Severe	11 (37)	12 (40)	7 (23)	0.029
Smoking status	Never	22 (30)	30 (40)	22 (30)	
	Ex	26 (41)	17 (27)	21 (33)	
	Current	9 (27)	11 (33)	13 (39)	0.64
Tumour site	Colon	36 (31)	40 (35)	39 (34)	
	Rectum	23 (39)	19 (32)	17 (29)	0.33
T stage	T 1/2	8 (24)	9 (27)	16 (49)	
	T 3	35 (37)	32 (34)	27 (29)	
	T 4	16 (34)	18 (38)	13 (28)	0.08
N stage	N 0	35 (33)	34 (32)	36 (34)	
	N 1	18 (38)	17 (35)	13 (27)	
	N 2	6 (29)	8 (38)	7 (33)	0.85
TNM stage	Stage I	6 (22)	7 (26)	14 (52)	
	Stage II	29 (38)	26 (34)	22 (29)	
	Stage III	24 (34)	26 (37)	20 (29)	0.14
Venous invasion	Absent	24 (31)	28 (36)	25 (33)	
	Present	35 (36)	31 (32)	31 (32)	0.66
Differentiation	Well/mod	55 (34)	54 (33)	54 (33)	
	Poor	4 (36)	5 (46)	2 (18)	0.49
Lymph nodes retrieved	>12	42 (32)	47 (36)	41 (32)	
	<12	17 (39)	12 (27)	15 (34)	0.79

* *X* ^2^ linear-by-linear analysis.

**Figure 4:**
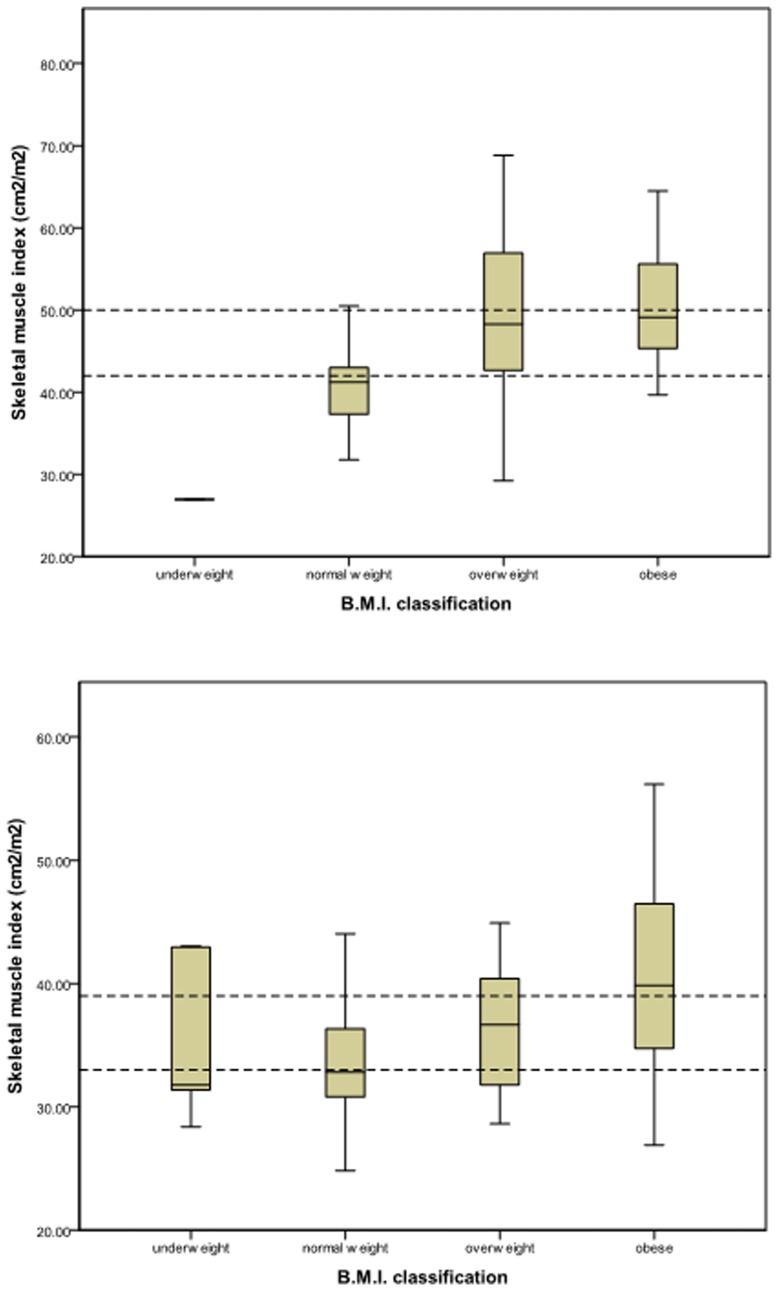
The relationship between B.M.I. classification and skeletal muscle index in male (top panel) and female (bottom panel) patients with primary operable colorectal cancer. Dashed lines represent cutoff values of the sex-specific tertiles.

The relationships between BMI classification and skeletal muscle index are illustrated in Figure 4. At least some patients from all the BMI categories fell within the lowest tertile of skeletal muscle index. In females, this meant a total of 24 patients (30%) with a normal, overweight or obese BMI were within the lowest tertile of skeletal muscle index. In males, 31 patients (33%) with a normal, overweight or obese BMI were within the lowest tertile of skeletal muscle index (Figure 4).

## Discussion

The results of the present study demonstrate a strong association between low skeletal muscle mass and the presence of a systemic inflammatory response, as measured by mGPS, in patients with primary operable colorectal cancer. Furthermore, there were no direct relationships between skeletal muscle mass and any tumour-related variables, including tumour stage or nodal status. Taken together, these results would suggest that the loss of lean tissue in cancer cachexia may be driven by the host systemic inflammatory response.

The negative impact of systemic inflammation on cancer outcome has been reported previously; associated with an increased risk of septic complications [24], functional decline and decreased survival [25]. The present study confirms that, using a different methodological approach, systemic inflammation plays a role in the development of muscle wasting in patients with colorectal cancer. This is supported by experimental models whereby pro-inflammatory cytokines, including interleukin-1 (IL-1), IL-6 and tumour necrosis factor-ά (TNF), have been reported as mediators of both anorexia and skeletal muscle proteolysis [15], the key components of weight loss in patients with cancer. Furthermore, the present study points to such inflammatory mediators having an effect on the liver, key to the elaboration of the systemic inflammatory response [26]. In addition to the hepatic production of acute phase proteins and their influence on skeletal muscle metabolism, there is also an increase in liver enzyme activity associated with an elevated mGPS [27], [28]. Overall, these results highlight the potential importance of a liver-derived systemic inflammatory response in the progressive nutritional and functional decline of patients with colorectal cancer. It should be emphasised that these findings may also be applicable to benign disease. Indeed, similar observations regarding the depletion of skeletal muscle being associated with activation of the systemic inflammatory response have been made in non-cancer cohorts, including patients with renal failure and chronic obstructive airways disease [11], [12].

Several previous studies investigating the clinical impact of cancer cachexia have focused specifically on the loss of lean tissue [4], [29]. However, in cancer patients, muscle wasting can occur with or without the loss of adipose tissue while in non-cancer patients there is evidence that obesity and visceral adipose tissue in particular are associated with a low grade inflammatory state [30], [31]. In order to examine these relationships in detail we included measures of both adipose tissue and skeletal muscle and can now report that a systemic inflammatory response in patients with colorectal cancer is associated with a reduction in skeletal muscle as opposed to an increase in visceral adiposity.

It is clear from the present study that a simple measure of BMI is insufficient to detect the changes in body composition associated with malignant disease. This is particularly true in populations with an increasing prevalence of obesity; it is of interest that only 3% of patients in the present study were classified as underweight according to WHO classification. Even the application of a cutoff value of <20, as suggested by Fearon and co-workers [10] as a more sensitive indicator of cachexia, increased this figure to only 5%. It is evident that traditional descriptors of body composition, such as BMI, do not have the capacity to adequately identify patients with reduced levels skeletal muscle [32]. The present study, therefore, supports the use of cross-sectional imaging to assess the body composition of patients with malignant disease [33]. By comparing two widely-available software packages, we have demonstrated that such analysis of CT scans is an objective and reproducible method of quantifying body composition.

In the present study we chose to use sex-specific tertiles rather than specific cutoff values to define levels of adiposity and sarcopenia. The most common current definition of sarcopenia is an appendicular skeletal muscle index more than two SDs below that of healthy adults (5.45 kg/m2 for females and 7.26 kg/m2 for males) [34]. These values relate to dual-energy x-ray (DEXA) scanning and may not be readily applied to cross-sectional imaging. Prado and co-workers, using CT image analysis, defined a skeletal muscle index of 52.4 cm^2^/m^2^ in men and 38.5 cm^2^/m^2^ in women as associated with mortality [35]. However, the population on which these cutoff values were developed was highly selective, consisting of 250 patients with an obese BMI (≥30) and a heterogeneous selection of respiratory tract and gastrointestinal cancers. Application of these cutoff values to the present cohort would have resulted in over 70% of patients being classified as ‘sarcopenic’; a figure which highlights the need for additional reference values for cross-sectional imaging modalities. Indeed, an international consensus group on the diagnostic criteria for cancer cachexia concluded that definitive cutoffs for the diagnosis of sarcopenia still need to be determined from large contemporary datasets [10].

This study has a number of limitations. Height and weight data were primarily based on patient-reported values, although these have proven reliable in previous studies [36], [37]. Electronic records of CT images were difficult to access prior to 2006 and only routinely available after 2008, meaning long term outcomes could not be assessed. In addition, although cancer-related weight loss is a continuous process, this study only assessed body composition at a single point in time. The changes in adipose tissue and skeletal muscle mass which occur over time and the relationships with cancer survival are of considerable interest and will be the subject of future work.

The present study adds important objective evidence to what is often empirically accepted; that patients with cancer preferentially lose lean tissue during the cachectic process. In addition, these results highlight a direct relationship between low levels of skeletal muscle and the presence of a systemic inflammatory response in patients with primary operable colorectal cancer.

## References

[1] Bosanquet N, Sikora K (2004) The economics of cancer care in the UK. Lancet Oncol 5(9): 568–574. 15337487 10.1016/S1470-2045(04)01569-4

[2] Dewys WD, Begg C, Lavin PT, Band PR, Bennett JM, et al (1980) Prognostic effect of weight loss prior to chemotherapy in cancer patients. Eastern Cooperative Oncology Group. Am J Med 69(4): 491–497. 7424938 10.1016/s0149-2918(05)80001-3

[3] Khalid U, Spiro A, Baldwin C, Sharma B, McGough C, et al (2007) Symptoms and weight loss in patients with gastrointestinal and lung cancer at presentation. Support Care Cancer 15(1): 39–46. 16786329 10.1007/s00520-006-0091-0

[4] Peng PD, van Vledder MG, Tsai S, de Jong MC, Makary M, et al (2011) Sarcopenia negatively impacts short-term outcomes in patients undergoing hepatic resection for colorectal liver metastasis. HPB (Oxford) 13(7): 439–446. 21689226 10.1111/j.1477-2574.2011.00301.x PMC3133709

[5] Ross PJ, Ashley S, Norton A, Priest K, Waters JS, et al (2004) Do patients with weight loss have a worse outcome when undergoing chemotherapy for lung cancers? Br J Cancer 90(10): 1905–1911. 15138470 10.1038/sj.bjc.6601781 PMC2409471

[6] Prado CM, Baracos VE, McCargar LJ, Mourtzakis M, Mulder KE, et al (2007) Body composition as an independent determinant of 5-fluorouracil-based chemotherapy toxicity. Clin Cancer Res 13(11): 3264–3268. 17545532 10.1158/1078-0432.CCR-06-3067

[7] O’Gorman P, McMillan DC, McArdle CS (2000) Prognostic factors in advanced gastrointestinal cancer patients with weight loss. Nutr Cancer 37(1): 36–40. 10965517 10.1207/S15327914NC3701_4

[8] Andreyev HJ, Norman AR, Oates J, Cunningham D (1998) Why do patients with weight loss have a worse outcome when undergoing chemotherapy for gastrointestinal malignancies? Eur J Cancer 34(4): 503–509. 9713300 10.1016/s0959-8049(97)10090-9

[9] van Vledder MG, Levolger S, Ayez N, Verhoef C, Tran TC, et al (2012) Body composition and outcome in patients undergoing resection of colorectal liver metastases. Br J Surg 99(4): 550–557. 22246799 10.1002/bjs.7823

[10] Fearon K, Strasser F, Anker SD, Bosaeus I, Bruera E, et al (2011) Definition and classification of cancer cachexia: an international consensus. Lancet Oncol 12(5): 489–495. 21296615 10.1016/S1470-2045(10)70218-7

[11] Kotler DP (2000) Cachexia. Ann Intern Med 133(8): 622–634. 11033592 10.7326/0003-4819-133-8-200010170-00015

[12] Morley JE, Thomas DR, Wilson MM (2006) Cachexia: pathophysiology and clinical relevance. Am J Clin Nutr 83(4): 735–743. 16600922 10.1093/ajcn/83.4.735

[13] Proctor MJ, Morrison DS, Talwar D, Balmer SM, O’Reilly DS, et al (2011) An inflammation-based prognostic score (mGPS) predicts cancer survival independent of tumour site: a Glasgow Inflammation Outcome Study. Br J Cancer 104(4): 726–734. 21266974 10.1038/sj.bjc.6606087 PMC3049591

[14] McMillan DC (2009) Systemic inflammation, nutritional status and survival in patients with cancer. Curr Opin Clin Nutr Metab Care 12(3): 223–226. 19318937 10.1097/MCO.0b013e32832a7902

[15] Argiles JM, Busquets S, Lopez-Soriano FJ (2005) The pivotal role of cytokines in muscle wasting during cancer. Int J Biochem Cell Biol 37(10): 2036–2046. 16105746 10.1016/j.biocel.2005.03.014

[16] McMillan DC, Scott HR, Watson WS, Preston T, Milroy R, et al (1998) Longitudinal study of body cell mass depletion and the inflammatory response in cancer patients. Nutr Cancer 31(2): 101–105. 9770720 10.1080/01635589809514687

[17] Fleming ID CJ, Henson DE, Hutter RVP, Kennedy BJ, Murphy GP, et al., editors. ( AJCC 5th Edition Cancer Staging Manual. Philadelphia: Lippincott-Raven.

[18] Proctor MJ, Morrison DS, Talwar D, Balmer SM, Fletcher CD, et al (2011) A comparison of inflammation-based prognostic scores in patients with cancer. A Glasgow Inflammation Outcome Study. Eur J Cancer 47(17): 2633–2641. 21724383 10.1016/j.ejca.2011.03.028

[19] Roxburgh CS, McMillan DC (2010) Role of systemic inflammatory response in predicting survival in patients with primary operable cancer. Future Oncol 6(1): 149–163. 20021215 10.2217/fon.09.136

[20] Maltoni M, Caraceni A, Brunelli C, Broeckaert B, Christakis N, et al (2005) Prognostic factors in advanced cancer patients: evidence-based clinical recommendations–a study by the Steering Committee of the European Association for Palliative Care. J Clin Oncol 23(25): 6240–6248. 16135490 10.1200/JCO.2005.06.866

[21] Walsh SR, Cook EJ, Goulder F, Justin TA, Keeling NJ (2005) Neutrophil-lymphocyte ratio as a prognostic factor in colorectal cancer. J Surg Oncol 91(3): 181–184. 16118772 10.1002/jso.20329

[22] McMillan DC (2008) An inflammation-based prognostic score and its role in the nutrition-based management of patients with cancer. Proc Nutr Soc 67(3): 257–262. 18452641 10.1017/S0029665108007131

[23] Mitsiopoulos N, Baumgartner RN, Heymsfield SB, Lyons W, Gallagher D, et al (1998) Cadaver validation of skeletal muscle measurement by magnetic resonance imaging and computerized tomography. J Appl Physiol 85(1): 115–122. 9655763 10.1152/jappl.1998.85.1.115

[24] Moyes LH, Leitch EF, McKee RF, Anderson JH, Horgan PG, et al (2009) Preoperative systemic inflammation predicts postoperative infectious complications in patients undergoing curative resection for colorectal cancer. Br J Cancer 100(8): 1236–1239. 19319134 10.1038/sj.bjc.6604997 PMC2676538

[25] Richards CH, Platt JJ, Anderson JH, McKee RF, Horgan PG, et al (2011) The impact of perioperative risk, tumor pathology and surgical complications on disease recurrence following potentially curative resection of colorectal cancer. Ann Surg 254(1): 83–89. 21572320 10.1097/SLA.0b013e31821fd469

[26] Gabay C, Kushner I (1999) Acute-phase proteins and other systemic responses to inflammation. N Engl J Med 340(6): 448–454. 9971870 10.1056/NEJM199902113400607

[27] Brown DJ, Milroy R, Preston T, McMillan DC (2007) The relationship between an inflammation-based prognostic score (Glasgow Prognostic Score) and changes in serum biochemical variables in patients with advanced lung and gastrointestinal cancer. J Clin Pathol 60(6): 705–708. 16644880 10.1136/jcp.2005.033217 PMC1955069

[28] Roxburgh CS, Wallace AM, Guthrie GK, Horgan PG, McMillan DC (2010) Comparison of the prognostic value of tumour- and patient-related factors in patients undergoing potentially curative surgery for colon cancer. Colorectal Dis 12(10): 987–994. 19555389 10.1111/j.1463-1318.2009.01961.x

[29] Pichard C, Kyle UG, Morabia A, Perrier A, Vermeulen B, et al (2004) Nutritional assessment: lean body mass depletion at hospital admission is associated with an increased length of stay. Am J Clin Nutr 2004 79(4): 613–618. 10.1093/ajcn/79.4.613 15051605

[30] Trayhurn P, Wood IS (2004) Adipokines: inflammation and the pleiotropic role of white adipose tissue. Br J Nutr 92(3): 347–355. 15469638 10.1079/bjn20041213

[31] Saijo Y, Kiyota N, Kawasaki Y, Miyazaki Y, Kashimura J, et al (2004) Relationship between C-reactive protein and visceral adipose tissue in healthy Japanese subjects. Diabetes Obes Metab 6(4): 249–258. 15171748 10.1111/j.1462-8902.2003.0342.x

[32] Thibault R, Genton L, Pichard C (2012) Body composition: Why, when and for who? Clin Nutr. 10.1016/j.clnu.2011.12.011 22296871

[33] Thibault R, Pichard C (2012) The evaluation of body composition: a useful tool for clinical practice. Ann Nutr Metab 60(1): 6–16. 22179189 10.1159/000334879

[34] Baumgartner RN, Koehler KM, Gallagher D, Romero L, Heymsfield SB, et al (1998) Epidemiology of sarcopenia among the elderly in New Mexico. Am J Epidemiol 147(8): 55–763. 10.1093/oxfordjournals.aje.a009520 9554417

[35] Prado CM, Lieffers JR, McCargar LJ, Reiman T, Sawyer MB, et al (2008) Prevalence and clinical implications of sarcopenic obesity in patients with solid tumours of the respiratory and gastrointestinal tracts: a population-based study. Lancet Oncol 9(7): 629–635. 18539529 10.1016/S1470-2045(08)70153-0

[36] Stunkard AJ, Albaum JM (1981) The accuracy of self-reported weights. Am J Clin Nutr 34(8): 1593–1599. 7270483 10.1093/ajcn/34.8.1593

[37] Perry GS, Byers TE, Mokdad AH, Serdula MK, Williamson DF (1995) The validity of self-reports of past body weights by U.S. adults. Epidemiology 6(1): 61–66. 7888448 10.1097/00001648-199501000-00012

